# Usefulness of handheld ultrasound devices in the assessment of abdominal pathology and comparison with high-end ultrasound devices

**DOI:** 10.1186/s13089-025-00433-5

**Published:** 2025-08-05

**Authors:** Ana Segura-Grau, Ines Salcedo-Joven, Esther Montes-Belloso, Sergio Cinza-Sanjurjo, Antonio Segura-Fragoso, Elena Segura-Grau

**Affiliations:** 1 Family and Community Medicine, Ultrasound Unit, San Francisco de Asis Hospital, Madrid, Spain; 2 Family and Community Medicine, Korea Strait Health Centre, Health Area of Madrid, Madrid, Spain; 3 Family and Community Medicine, Isabel II Health Centre, Health Area of Parla, Parla, Spain; 4 Family and Community Medicine, Milladoiro, Health Centre, Health Area, A Coruña, Spain; 5 Institute for Health Research of Santiago de Compostela (IDIS), Santiago de Compostela, Spain; 6 Biomedical Research Networking Centre, Centre-Cardiovascular Diseases(CIBERCV), Santiago de Compostela, Spain; 7 Department of Medicine, University of Santiago de Compostela, Santiago de Compostela, Spain; 8 Epidemiology Unit, Semergen’s Research Agency, Madrid, Spain; 9 Hospitalar ULS Viseu Dao Lafoes, Madrid, Portugal; 10 Semergen’s Ultrasound Working Group, Madrid, Spain

**Keywords:** Abdominal ultrasound, Portable devices, POCUS, Agreement

## Abstract

**Background and objective:**

The EFSUMB recommends the use of handheld ultrasound devices in many point-of-care cases, including primary care. However, it is necessary to continue training in conventional ultrasound examinations. Our aim is to analyze the diagnostic accuracy of handheld ultrasound devices in abdominal pathology compared with conventional high-end ultrasound scanners.

**Methodology:**

Agreement study between two ultrasound techniques, (1) POCUS (*point-of-care ultrasound)*, with a General Electric^®^ Vscan Air device and (2) standard ultrasound with a high-end Samsung RS 80^®^ ultrasound scanner. Cohen’s kappa was used for the analysis. The study was conducted between November 2022 and September 2023 in the general ultrasound unit of the San Francisco de Asís University Hospital in Madrid. It included all patients from the emergency department and the inpatient unit who have been referred for abdominal ultrasound.

**Results:**

A total of 93 patients were included (52.7% were women and the mean age was 65.6 (23.6) years). As regards body mass index (BMI), 11.8% had a BMI over 30 kg/m^2^. Of the scans performed, 69.9% were abdominal and the rest urological. Overall, the degree of agreement between the two tests was 89%, with 100% for liver and bladder pathology, 86.2% for renal pathology and 82.5% for complicated renal pathology. Intestinal (73.3%) and pancreatic (58.1%) pathologies showed the lowest correlation.

**Conclusions:**

The degree of agreement of handheld devices is high (89%), especially in renal and bladder pathologies, where ultrasound is decisive in decision-making. The agreement is weaker in pancreatic and gastrointestinal tract pathologies.

**Supplementary Information:**

The online version contains supplementary material available at 10.1186/s13089-025-00433-5.

## Introduction

Ultrasound imaging, with more than a century of history, has evolved significantly from the first static devices to modern ultraportable devices with artificial intelligence and digitalization. Its use has been crucial to improving physicians’ problem-solving capacity, allowing rapid and accurate clinical decision-making. The incorporation of handheld ultrasound scanners has transformed not only immediate diagnosis, but also longitudinal follow-up of patients in various settings such as home care, out-of-hospital emergency care and palliative care. This progress has reduced reliance on specialized services, optimizing resources and improving the quality of patient care [1].

Handheld ultrasound devices (HUDs) are playing an increasingly important role in the diagnosis and management of digestive pathologies. Their usefulness compared to high-end scanners is especially remarkable in contexts where portability, accessibility and rapid assessment are essential. Their compact design and light weight make them ideal for use in emergency settings, rural areas and home visits. Likewise, their affordable cost makes them a viable alternative for screening specific pathologies, such as abdominal aortic aneurysm, or for monitoring certain diseases [2,3].

Many factors can influence the quality of an ultrasound examination. In addition to factors that depend on the sonographer, e.g. their level of training, and those that depend on the patient’s characteristics, e.g. body mass index in abdominal ultrasound, other factors depend on the technical quality of the ultrasound device used [4].

The available literature highlights that adequate training is key to maximizing the usefulness of HUDs. Although these devices are promising, their accuracy and reliability diminish in more complex abdominal pathologies when the operator lacks the necessary training. This underlines the importance of training professionals in the effective use of these devices in different clinical settings [5].

The growing trend to equip primary care teams with conventional ultrasound machines, and increasingly with HUDs, in both urban and rural areas, stresses the need to evaluate the validity of these devices for patient assessment.

The main objective of this study was to analyze the diagnostic accuracy of handheld ultrasound devices in bedside assessment of abdominal pathology compared to conventional high-end ultrasound devices, taking into account the final clinical diagnosis.

## Materials and methods

### Study design

Validation study comparing two ultrasound techniques, conducted during routine clinical practice.

It was carried out in the general ultrasound unit of the San Francisco de Asís University Hospital in Madrid, between November 2022 and September 2023. All measures were obtained by a family doctor with more than 15 years of experience in the central imaging department of this hospital.

All the patients recruited underwent two ultrasound examinations by the same technician: (1) first, a POCUS (*point-of-care ultrasound)* with a General Electric^®^ Vscan Air, a test which is performed in a short period of time and must answer a specific clinical question; and (2) standard ultrasound in the general ultrasound unit, with no time limit, using a high-end Samsung RS 80^®^ ultrasound scanner. This second examination was considered the reference standard or gold standard, and was the test used to establish the final diagnosis. Both tests were done at the same time, first the POCUS and second the standard ultrasound. This sequence prevents the physician from having any reliable information during the POCUS examination, but not in the standard ultrasound. Our design avoids bias related to inter-observer variability and also avoids any advantage for the HUD, which is the device that we were evaluating.

This study was approved by the Ethics Committee for Investigation with Medicinal products of the Princesa University Hospital, Madrid, with the registration code SEM_ECO_1_22, on 23-06-22.

### Patients

This research included all the patients from the emergency department and inpatient unit who underwent abdominal ultrasound during the study period, and who were referred to the Principal Investigator’s office.

Patients who did not sign the informed consent form and those who had the two scans performed by different technicians were excluded.

The sample size required to assess agreement between two observer-dependent diagnostic imaging tests was calculated, ensuring that same professional performed all the examinations. This significantly minimizes observation bias. A sample size of more than 100 patients was considered sufficient to ensure adequate statistical power to confirm agreement between the two techniques. The sample size was estimated based on the prevalence of each group of diseases considering the prevalence of each disease [6, 7], as explained in the following section.

Based on this estimate, the recruitment period was limited to six months. According to the previous year’s records (with an average of 300 requests for abdominal ultrasound per year), this interval was considered sufficient to reach the required sample and also to avoid temporal biases associated with longer recruitment periods.

### Diagnostic criteria

The diagnosis of the different abdominal pathologies was made according to the following criteria:

- Hepatomegaly: a longitudinal axis of the right liver lobe greater than 15.5 cm at a midclavicular longitudinal section [8].
- Hepatic steatosis: diffusely increased hepatic echogenicity with posterior attenuation and loss of definition of the intra-hepatic vessels [8].
- Cirrhosis was suspected if there was coarse-grained structure, nodular surface, hypertrophy of the caudate lobe, spleno-portal axis abnormalities, portal vein greater than 13 mm, and presence of indirect signs such as ascites or collateral circulation [8].
- Splenomegaly: greater than 12 cm at the longitudinal axis [9].
- Gallbladder pathology: enlarged gallbladder when the transverse axis was greater than 4 cm and the wall was thickened more than 3 mm. The bile duct should be < 8 mm [6].
- Pancreas: normal size between 12.5 and 20 cm [7].

### Data analysis

Categorical data were presented as absolute frequency and percentage (%). Age was summarized as mean, standard deviation, minimum and maximum.

The diagnoses were grouped for the analysis by anatomical regions, considering the following classification:

- HEPATIC PATHOLOGY: hepatomegaly, space-occupying lesions, signs of diffuse liver disease.
- INTESTINAL PATHOLOGY: signs of appendicitis, signs of diverticulitis, signs of intestinal obstruction.
- PANCREATIC PATHOLOGY: enlargement, space-occupying lesions, altered echogenicity.
- RENAL PATHOLOGY: hydronephrosis, renal lithiasis, solid lesions.
- COMPLICATED RENAL PATHOLOGY: urinoma, pyonephrosis, signs of pyelonephritis, abscess.
- BLADDER PATHOLOGY: polyp, lithiasis, trabeculated bladder, clot.
- GALLBLADDER AND BILIARY PATHOLOGY: enlargement, wall thickening, lithiasis, biliary sludge, intra- or extrahepatic bile duct dilatation.
- OTHERS: aortic pathology, spleen pathology, ascites, pleural effusion, prostate pathology.

### Normal result

IBM SPSS Statistics version 23 was used for statistical analyses. The Forest Plot was made with Review Manager 5.4.1.

### Outcomes

The positive and negative predictive values, and the sensitivity and specificity of POCUS were calculated in comparison with standard ultrasound, which was the gold standard. A Forest Plot was designed with the sensitivities and specificities of the different pathologies and their 95% confidence intervals (95% CI).

Cohen’s kappa was used to assess the agreement between the two ultrasound techniques. Values 0.00 to 0.20 were interpreted as no or slight agreement, 0.21 to 0.40 as fair agreement, 0.41 to 0.60 as moderate agreement, 0.61 to 0.80 as substantial agreement, and 0.81 to 1.00 as almost perfect agreement.

In the hypothesis test, the null hypothesis was rejected in comparisons with p values < 0.05.

### Results

A total of 93 patients were recruited, of which 52.7% were women. The mean age was 65.6 (23.6) years, with 38.7% over 80 and 36.6% under 60. As regards body mass index (BMI), 11.8% had a BMI greater than 30 kg/m^2^ and 14.0% had a BMI < 20 kg/m^2^.

**Fig. 1:**
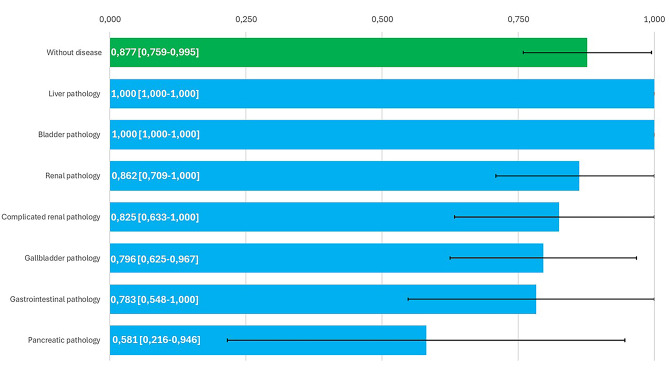
Kappa indices for each group of pathologies

Of the examinations performed, 69.9% were abdominal and the rest were urological. By anatomical systems, renal pathology was the most prevalent (23.6%, *n* = 22), followed by gallbladder and biliary pathology (14%, *n* = 13), bladder pathology (9.7%, *n* = 9) and gastrointestinal pathology (9.7%, *n* = 9). Liver pathology accounted for 6.5% (*n* = 6) of diagnoses, other pathological findings were 8.6% (*n* = 8) of cases and 20.4% (*n* = 19) were normality cases. The other pathological findings included ascites (*n* = 2), prostatic hypertrophy (*n* = 2), abdominal aortic aneurysm (*n* = 1), pelvic mucocele (*n* = 1), splenic cyst (*n* = 1) and pleural effusion (*n* = 1). Figure 1 shows agreement for diagnosis of the different pathologies by system.

**Fig. 2:**
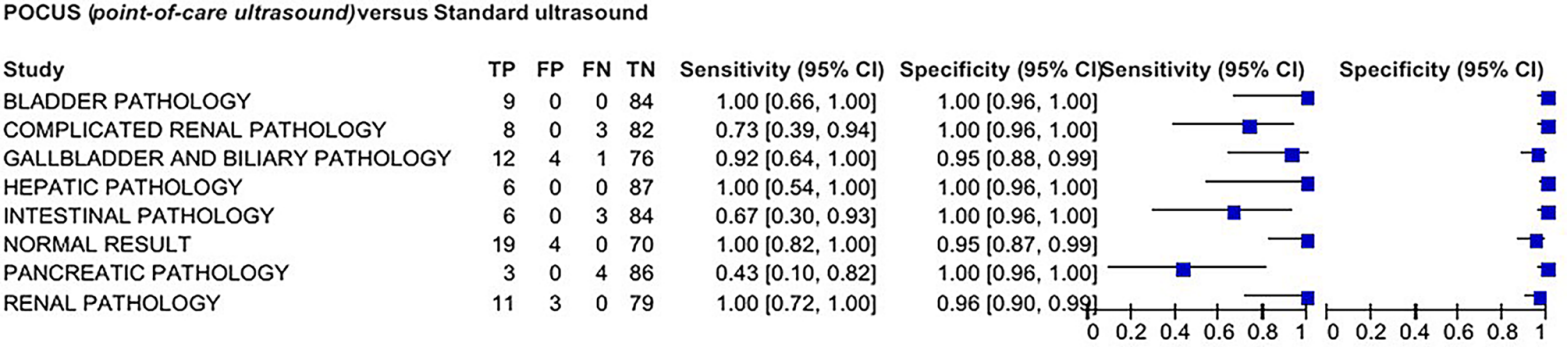
Forest Plot of sensitivity and specificity for each of the pathologies. TP: true positive; FP: false positive; TN: true negative; FN: false negative. 95% CI: 95% confidence interval

A diagnostic agreement of 89% was found between HUDs and high-end ultrasound devices. Figure 2 represents sensitivity (Sn) and specificity (Sp) for abdominal pathologies.

The 11 cases in which agreement between the two devices was not detected corresponded to the following pathologies: 3 (27.3%) to gastrointestinal tract disorders (diverticulitis, inguinal hernia), 3 (27.3%) to pancreatic pathology (mild pancreatitis), 3 (27.3%) to complicated renal pathology (pyonephrosis, pyelonephritis and complex cyst), 1 (9%) to gallbladder pathology (polyp) and 1 (9%) to ascites.

## Discussion

The results obtained from a sample of patients with abdominal symptoms undergoing abdominal ultrasound by the same observer show a strong agreement between handheld and high-end devices (89%) in the diagnosis of the most prevalent pathologies. These include aortic aneurysms, urological pathologies (hydronephrosis, renal and bladder lithiasis, bladder globe) and biliary diseases, especially relevant in primary care and emergency settings. This degree of agreement may make HUDs recommendable as a complement to physical examination, particularly in pathologies with a higher agreement. In contrast, a poorer agreement was found in pancreatic and intestinal pathologies, which are more difficult to diagnose by ultrasound. These often require higher quality imaging devices (e.g. high-end ultrasound scanners) and other diagnostic tests (e.g. lab tests or other imaging tests) to reach a definitive diagnosis and establish a therapeutic plan.

After reviewing the available literature, this is the first time that the diagnostic accuracy of a handheld ultrasound device has been analyzed in the clinical context of abdominal pain. This symptom is highly prevalent in PC and causes numerous consultations, since around 85% of patients with this clinical symptom consult in PC [10]. The use of handheld devices in PC, together with anamnesis and physical examination, will allow for better diagnostic orientation. This is because their performance is similar to high-end devices, but faster for the patient and more efficient for the health system, avoiding delays and unfruitful interventions.

The approach to acute abdominal pain in primary care [11] is frequently associated with aortic aneurysm, cholecystitis, and reno-ureteral colic. These conditions have easily identifiable ultrasound characteristics, even with HUDs, which reinforces their usefulness for these cases in PC. Likewise, HUDs are also proving to be very useful in the assessment of urological pathology, particularly in the initial approach to prevalent symptoms like hematuria. This clinical sign can have multiple causes -from renal lithiasis to neoplastic processes- and requires an accurate diagnostic approach to determine its etiology and rule out malignant conditions [12].

Conversely, we found a lower degree of agreement in more complex pathologies, like pancreas and gastrointestinal tract diseases. This is an important limitation of HUDs, which have lower resolution and diagnostic accuracy in these locations where ultrasound is more complex. In the case of the pancreas, its retroperitoneal location and relative depth require greater penetrability and better resolution. Thus, its assessment is more difficult and requires better image quality. The examination of the gastrointestinal tract is particularly challenging due to intestinal meteorism, which interferes with ultrasound propagation, and to the depth of the intestinal loops, which makes a thorough evaluation difficult. This is consistent with the available literature, which describes the greater diagnostic complexity of pathologies like appendicitis and acute diverticulitis due to factors such as meteorism and intestinal contents that limit adequate visualization of the affected structures [11].

This reinforces the need to complement the findings of HUDs with conventional high-end ultrasound in more complex cases, as noted by Alfuraih et al. [2].

The scarce literature on ultraportable ultrasound devices in abdominal pathology reflects the limited evidence conclusively supporting their validity in daily clinical practice [4]. The increasing use of HUDs is due to advantages such as convenience or affordability, but it has not been supported by solid scientific evidence of their diagnostic accuracy. The European Federation of Societies for Ultrasound in Medicine and Biology (EFSUMB) published a position paper in this regard that highlights the usefulness of handheld ultrasound devices at the point of care of acute patients. However, it emphasizes that their use should be targeted and well-defined, and underlines the need for further research to strengthen the evidence, which remains limited [13]. Our work provides results to this knowledge gap and confirms the diagnostic correlation with high-end devices in pathologies that frequently cause abdominal pain. However, it casts doubts about pathologies of organs that are more difficult to examine sonographically, such as the pancreas and intestine.

Clinical ultrasound, from its origins to the present day, has proven to be an invaluable tool in healthcare, especially in settings such as primary care. Technological advances in ultraportable ultrasound have significantly transformed its role in clinical practice, broadening its scope and consolidating it as an essential tool at the point of patient care (POCUS). Despite limitations in the diagnosis of certain diseases, HUDs stand out as accessible, practical and cost-effective tools for the initial evaluation and follow-up of some abdominal pathologies. The remarkable development of these devices in recent years has resulted in a significant increase in image quality, functionality and diagnostic accuracy. Therefore, previous studies are not comparable with current research due to the constant improvement of their performance [14].

This development reinforces the need for updated studies that reflect their true potential, since these devices are becoming increasingly viable tools in primary care, emergency departments and rural areas, where patient mobility or availability of advanced equipment are limited. As Dr Rivera^1^ points out, the use of ultraportable devices with advanced features, e.g. digitalization and artificial intelligence, is transforming medical diagnosis by enabling fast and effective decision-making directly at the point of care. This not only improves immediate clinical problem-solving, but also the decision-making capacity of primary care physicians, reducing unnecessary referrals and optimizing healthcare resources. In this sense, López et al. have described them as the ‘stethoscope of the 21st century’, emphasizing their growing relevance in modern clinical practice [15]. The technological development of ultraportable ultrasound devices used by trained physicians enhances problem-solving and decision-making in primary care. This allows reducing delays in imaging departments and implementing treatments in a shorter time, which improves the prognosis of patients.

Although our results were obtained using a rigorous methodology and appropriate statistical analyses, our work is not without limitations. These include the lack of previous clinical information on the patients, which would probably improve the diagnostic accuracy of ultrasound. However, this does not imply a bias because it affects both techniques equally, which minimizes its impact on the comparison. In addition, the sequence of the examinations (POCUS followed by high-end ultrasound) might have favored the second scan by providing prior diagnostic guidance. However, this would only affect the correlation of the POCUS, which suggests that in a real clinical setting its performance might be even better.

It should also be noted that ultrasound is an observer-dependent test. Therefore, any analysis of correlation or diagnostic performance could provide discrepant results depending on the observer. In our study, the same observer performed both tests on each patient. This reduced variability and avoided biases derived from unequal information between observers, which becomes a strength rather than a limitation. As a consequence of the design of our study, we do not know inter-observer variability in order to analyze the correlation between the two devices. In addition, performing the HUDs scan first reduces the possibility of bias in favor of this device, but it does not avoid prior information when the physician did the examination with the high-end ultrasound. Finally, our research does not analyze specific diagnoses, but rather pathologies by systems. This methodological choice seeks to avoid underrepresentation of infrequent pathologies and to ensure statistical power. This grouping is more appropriate, because diagnostic difficulty in ultrasound has more to do with the anatomical region than with the specific disease.

## Conclusions

Our results confirm that the diagnostic accuracy of handheld ultrasound devices in POCUS examinations of abdominal pathologies is very high, with a diagnostic correlation of 89% with respect to complete and standard examinations with high-end ultrasound devices. HUDs proved to be most efficient and cost-effective in pathologies in which ultrasound becomes decisive in decision-making, like urological (renal and bladder) and biliary pathologies. On the other hand, their usefulness was more limited in pancreatic and gastrointestinal tract pathologies, because, although ultrasound can play an important role, clinical, analytical and/or other imaging techniques are more relevant in these cases.

While high-end ultrasound devices remain essential for more detailed and complex assessments, ultraportable devices offer an acceptable alternative to increase the clinician’s diagnostic capacity in contexts where swiftness and accessibility are crucial. Moreover, ultraportable devices may be useful to complement physical examination and clinical decision-making in a short time, improving patient care. However, there are important limitations in its diagnostic accuracy in pancreatic and gastrointestinal tract pathologies.

**Table 1:** Socio-demographic and clinical characteristics

Variable	% (*n*)
N	93
Women, % (n)	52.7% (49)
Age, mean [SD]	65.6 [23.6]
< 60 years, % (n)	36.6% (34)
60–79 years, % (n)	24.7% (23)
≥ 80 years, % (n)	38.7% (36)
BMI	
< 20 kg/m^2^, % (n)	14.0% (13)
20–30 kg/m^2^, % (n)	74.2% (69)
> 30 kg/m^2^, % (n)	11.8% (11)
Type of ultrasound exam	
Abdominal, % (n)	67.7% (63)
Urological, % (n)	30.1% (28)
Abdominal-Pelvic, % (n)	2.2% (2)

SD: standard deviation; BMI: Body mass index

## Electronic supplementary material

Below is the link to the electronic supplementary material.

Supplementary Material 1

## Data Availability

The data would be available after a reasonable request to the scientific committee.
