# Supplementary material for: Usefulness of handheld ultrasound devices in the assessment of abdominal pathology and comparison with high-end ultrasound devices
Source: Ultrasound J. 2025 Aug 5;17:38. doi: 10.1186/s13089-025-00433-5 (PMC12325146; doi:10.1186/s13089-025-00433-5)
Supplement: Supplementary file 1 — Supplementary Material 1 [file 13089_2025_433_MOESM1_ESM.pdf]

COMITÉ DE ÉTICA DE LA INVESTIGACIÓN CON MEDICAMENTOS
ACTA CEIm NÚMERO 10/22
26/05/2022

TITULO: Utilidad de los ecógrafos de bolsillo en la valoración de la patología abdominal y comparación con ecógrafos de alta gama: experiencia
TIPO: Estudio observacional (LIB 14/2007)
CODIGO: SEM_ECO_1_22
Nº EudraCT: N/A
Nº de Registro: 4881
Investigador Principal: Ana Segura Grau (Hospital Universitario San Francisco de Asís, Madrid)
Promotor: N/A
CEIm: Hospital Universitario La Princesa
DECISIÓN TOMADA: Solicitud de aclaraciones (26-05-22, acta CEIm 10/22)
1. Incluir nombre del promotor e indicar si existe financiación.
2. Se debe justificar el tamaño muestral (100 o 300 sujetos)
3. Detallar la variable con la que se va a conseguir demostrar el objetivo principal y correlacionar los datos ecográficos con los clínicos.
4. Incluir cómo se va a realizar el tratamiento de los datos (codificación, anonimización...)
5. Unificar si la ecografía será abdominal o urológica o ambas.
6. En la HIP/CI se deben hacer los siguientes cambios:
a. Corregir la siguiente frase: "la información recogida ayudará a un mejor" y ponerla en condicional "podría mejorar"

DOCUMENTOS EVALUADOS:
- Protocolo - Versión 1, 26 de enero de 2022
- HIP/CI - Versión 1, 26 de enero de 2022

Nota: Se debe enviar documento con la Respuesta a las Aclaraciones, así como las versiones con y sin control de cambios de los documentos modificados.
